# Genome-Wide Identification, Evolution, and Expression Analysis of the *DMP* Gene Family in Peanut (*Arachis hypogaea* L.)

**DOI:** 10.3390/ijms26157243

**Published:** 2025-07-26

**Authors:** Pengyu Qu, Lina He, Lulu Xue, Han Liu, Xiaona Li, Huanhuan Zhao, Liuyang Fu, Suoyi Han, Xiaodong Dai, Wenzhao Dong, Lei Shi, Xinyou Zhang

**Affiliations:** 1 School of Life Sciences, Zhengzhou University, Zhengzhou 450001, China; 17837860522@163.com (P.Q.); he19031@foxmail.com (L.H.); fuly@zzu.edu.cn (L.F.); 2 Institute of Crop Molecular Breeding, Henan Academy of Agricultural Sciences/Key Laboratory of Oil Crops in Huang-Huai-Hai Plains, Ministry of Agriculture/Henan Provincial Key Laboratory for Oil Crops Improvement/National and Provincial Joint Engineering Laboratory for Peanut Genetic Improvement/The Shennong Laboratory/National Invocation Center for Bio-Breeding Industry, Zhengzhou 450002, China; luluxue9331@163.com (L.X.); liu1856215@163.com (H.L.); li12568xiaona@163.com (X.L.); z2023huan@163.com (H.Z.); suoyi_han@126.com (S.H.); daixiaodong6666@163.com (X.D.); dongwzh@126.com (W.D.); 3 College of Agronomy, Shenyang Agricultural University, Shenyang 110866, China

**Keywords:** DMP, evolution, gene expression, haploid induction, peanut

## Abstract

Peanut (*Arachis hypogaea* L.) is a globally important oilseed cash crop, yet its limited genetic diversity and unique reproductive biology present persistent challenges for conventional crossbreeding. Traditional breeding approaches are often time-consuming and inadequate, mitigating the pace of cultivar development. Essential for double fertilization and programmed cell death (PCD), DUF679 membrane proteins (DMPs) represent a membrane protein family unique to plants. In the present study, a comprehensive analysis of the DMP gene family in peanuts was conducted, which included the identification of 21 family members. Based on phylogenetic analysis, these genes were segregated into five distinct clades (I–V), with *AhDMP8A*, *AhDMP8B*, *AhDMP9A*, and *AhDMP9B* in clade IV exhibiting high homology with known haploid induction genes. These four candidates also displayed significantly elevated expression in floral tissues compared to other organs, supporting their candidacy for haploid induction in peanuts. Subcellular localization prediction, confirmed through co-localization assays, demonstrated that AhDMPs primarily localize to the plasma membrane, consistent with their proposed roles in the reproductive signaling process. Furthermore, chromosomal mapping and synteny analyses revealed that the expansion of the *AhDMP* gene family is largely driven by whole-genome duplication (WGD) and segmental duplication events, reflecting the evolutionary dynamics of the tetraploid peanut genome. Collectively, these findings establish a foundational understanding of the *AhDMP* gene family and highlight promising targets for future applications in haploid induction-based breeding strategies in peanuts.

## 1. Introduction

Membrane proteins are integral to a wide array of biological processes, including cell differentiation, signal recognition, and the transport of molecules, especially during the intricate events of double fertilization. For instance, EC1 proteins, secreted by the egg cell, are essential for triggering sperm cell activation and promoting their separation, while *GEX2* regulates the recognition and adhesion between male and female gametes [1,2,3,4,5]. Among the plant-specific membrane-associated proteins, DUF679 membrane proteins (DMPs) represent a unique family, with DMP8 and DMP9 playing pivotal roles in gamete fusion [6]. In *Arabidopsis thaliana*, *AtDMP1* has been linked to plant senescence and is thought to function in membrane remodeling and the regulation of programmed cell death (PCD), potentially through its interactions with the endoplasmic reticulum (ER) and tonoplast [7]. *AtDMP3* and *AtDMP4* are highly expressed in senescing tissues, with *AtDMP4* co-expressing with *RIBONUCLEASE 3 (RNS3)* and *BIFUNCTIONAL NUCLEASE 1 (BFN1)*, which are both key regulators of developmental PCD (dPCD) [8]. Other members, such as *AtDMP2*, *AtDMP6*, and *AtDMP7,* exhibit broad expression across various tissues and organs. In contrast, *AtDMP8*, *AtDMP9*, and *AtDMP10* are specifically expressed in floral organs, with β-glucuronidase (GUS) histochemical assays showing that *AtDMP9* is localized predominantly in pollen grains [9]. Functional studies using RNA interference (RNAi) to target knockdown *AtDMP9*, combined with the high-resolution imaging of sperm cells, have demonstrated its critical role in the double fertilization process [10]. Additionally, in potato (*Solanum tuberosum* L.), *StDMP2* has been found to positively regulate resistance to *Phytophthora* infection [11].

A cross-species analysis of the *DMP* gene family, spanning from lower plants to angiosperms, revealed an evolutionary trajectory shaped predominantly by strong purifying selection, and accompanied by events of intron loss and gene loss [12]. In oats (*Avena sativa* L.), specific *AsDMP* genes have been implicated in regulating seed aging and longevity, highlighting the functional diversity of this gene family [13]. In cotton (*Gossypium* spp.), 58 *DMP* genes have been identified across four species, with evidence of functional divergence among certain paralogous pairs, particularly during fiber development, highlighting the complexity and potential biological significance of these genes [14]. Similarly, a genome-wide analysis in soybeans (*Glycine max* L.) identified 14 *GmDMP* genes, including two candidates implicated in haploid induction [15].

Peanut (*Arachis hypogaea* L., 2*n* = 4*x* = 40, AABB) is an allotetraploid species believed to have arisen through sexual hybridization and subsequent natural chromosome doubling between two diploid wild progenitors: *Arachis duranensis* (2*n* = 2*x* = 20, AA) and *Arachis ipaensis* (2*n* = 2*x* = 20, BB). As a crop of global economic importance, peanuts provide essential vegetable oil and dietary proteins for human consumption [16]. However, its distinct reproductive biology presents substantial obstacles to conventional breeding. Peanut flowers undergo self-pollination above ground, followed by the development of a specialized organ known as the “peg”, which drives the fertilized ovary into the soil where pod formation occurs [17]. This unique reproductive mechanism contributes to the crop’s limited genetic diversity, impedes artificial pollination, and results in low success rates in crossbreeding, thereby limiting the efficiency and scope of cultivar improvement [18]. To overcome these limitations and meet growing market demands, doubled haploid (DH) technology via in vivo haploid induction has emerged as a cutting-edge strategy for accelerating crop improvement. By enabling the rapid fixation of recombinant haploid lines within just two generations, this approach dramatically shortens breeding cycles and enhances selection efficiency [19]. However, implementing DH technology in peanuts remains exceptionally difficult due to the crop’s recalcitrance to haploid induction. To date, only a single successful case has been reported to achieve a through in vitro anther culture [20,21], underscoring the urgent need for alternative and more efficient haploid induction systems in this species. In maize (*Zea mays* L.), the mutation of the *ZmDMP* gene significantly increases the haploid induction rate [22]. Similarly, in *A. thaliana*, knockout of the homologous genes *AtDMP8* and *AtDMP9* has been shown to induce haploids effectively. These findings have catalyzed the development of DMP-based haploid induction systems across a range of crops, including potato (*S. tuberosum* L.), watermelon (*Citrullus lanatus* Thunb. Matsum. & Nakai), cucumber (*Cucumis sativus* L.), rapeseed (*Brassica napus* L.), cotton, alfalfa (*Medicago sativa* L.), and soybean [22,23,24,25,26,27,28,29,30]. Given the high degree of functional conservation of *DMP8* and *DMP9* across dicotyledonous species, the targeted mutagenesis of their peanut homologs holds strong potential for establishing a haploid induction system, offering a transformative new method for peanut breeding.

To explore this potential, we conducted a genome-wide identification and functional characterization of *DMP* genes in peanuts. The primary objective of this study is to uncover the candidate genes involved in haploid induction and to lay the molecular groundwork for implementing DH technology in peanut breeding. This work not only contributes to the understanding of the *DMP* gene family’s evolutionary and functional dynamics but also opens a new avenue for precision breeding in this economically important crop.

## 2. Results

### 2.1. The Characterization of the AhDMP Gene Family in Peanuts

To explore the evolutionary features of the *DMP* gene family in plants, we analyzed 14 representative species. Maize was included as a monocotyledonous model, while a diverse range of dicotyledonous species were chosen from key botanical families: *Solanum lycopersicum, Nicotiana. tabacum*, and *S. tuberosum* from the Solanaceae family; *A. thaliana* and *B. napus* from the Brassicaceae family; *C. lanatus* and *C. sativus* from the Cucurbitaceae family; *Gossypium hirsutum* from the Malvaceae family; and *A. hypogaea*, *G. max*, *M. sativa*, *A. duranensis*, and *A. ipaensis* from the Leguminosae family. Using amino acid sequences of ten previously reported DMPs in *A. thaliana* as queries, we conducted BLASTp searches to identify homologous proteins across the selected species. In *A. hypogaea* cv. YZ9102, 21 putative *DMP* genes were identified, while eight and seven were detected in its diploid progenitors, *A. duranensis* and *A. ipaensis*, respectively. The numbers of *DMP* genes identified in other species were as follows: *M. sativa* (9), *G. max* (13), *Z. mays* (12), *G. hirsutum* (20), *B. napus* (31), *S. lycopersicum* (6), *C. lanatus* (6), *C. sativus* (6), *S. tuberosum* (6), and *N. tabacum* (11).

Based on phylogenetic analysis, *AhDMP* genes were systematically designated with A or B suffixes to distinguish homologous gene pairs. To support future functional characterization, the key physicochemical properties of the encoded proteins were predicted, including amino acid sequence length, isoelectric point (pI), molecular weight (MW), the number of transmembrane domains (TMs), and predicted subcellular localization. The proteins varied in length from 188 to 289 amino acids, with molecular weights ranging from 20.30 to 31.35 kDa and pI values between 4.8 and 9.3. Most AhDMPs were predicted to contain three or four transmembrane domains, except for AhDMP7A and AhDMP7B, which each possessed only two domains. Subcellular localization predictions indicated that all 21 AhDMPs were localized to the plasma membrane (Table 1).

### 2.2. Phylogenetic Analysis of AhDMPs

**Figure 1:**
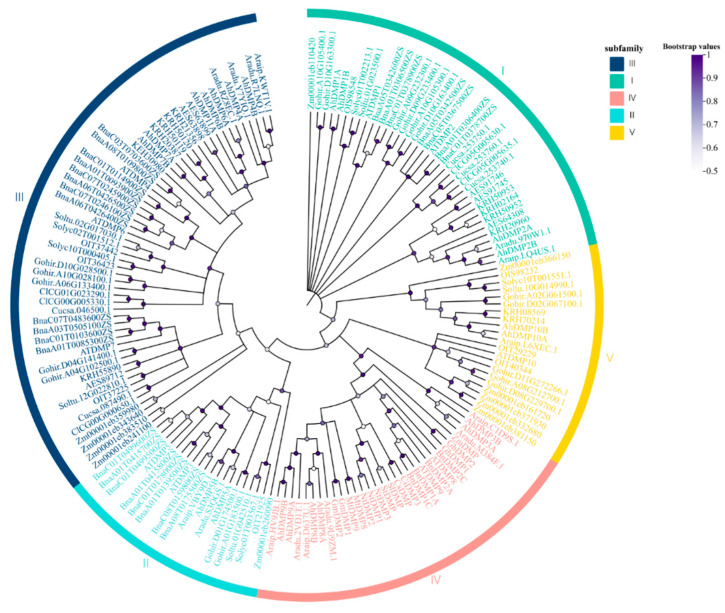
The phylogenetic analysis of the *DMP* gene family across 14 plant species using the Neighbor-Joining (NJ) method. Bootstrap analysis was performed with 1000 replicates to assess branch support. Different groups are distinguished by different colors. The scale and color bar indicate bootstrap confidence values ranging from 0.5 to 1.

A phylogenetic tree of 166 DMPs from 14 plant species was constructed using the Neighbor-Joining (NJ) method to investigate their evolutionary relationship. The resulting phylogenetic tree classified these genes into five distinct groups: Ⅰ, Ⅱ, Ⅲ, Ⅳ, and Ⅴ. Among them, Group Ⅲ contained the largest number of members (56), followed by Group Ⅳ (37), Group Ⅰ (32), Group Ⅴ (21), and Group Ⅱ (20) (Figure 1). The number of *DMP* genes in *A. hypogaea* exceeded that of its diploid progenitors. This increase may be attributed to gene duplication events or the retention of duplicated genes during tetraploidization, whereas some genes in the diploids may have undergone loss or pseudogenization. These observations suggest that *AhDMP* genes may contribute to the environmental adaptability of the tetraploid genome, with certain members potentially undergoing functional divergence through duplication and subfunctionalization.

Araip.D637U.1, Araip.HV9JB.1, and Araip.C1D9S.1 in *A. ipaensis*; Aradu.2VD1T.1, Aradu.9U9ZM.1, and Aradu.M384F.1 in *A. duranensis*; and AhDMP8A, AhDMP8B, AhDMP9A, AhDMP9B, AhDMP3A, and AhDMP3B in *A. hypogaea* were all clustered within Group IV of the phylogenetic tree. This clustering suggests a high degree of evolutionary conservation and the potential functional relevance of these genes. Interestingly, the three homologous gene pairs in *A. hypogaea*, AhDMP3A/B, AhDMP8A/B, and AhDMP9A/B, were found in the same phylogenetic group as several previously reported *DMP* genes known to induce haploids. These include ZmDMP (*Z. mays*), AtDMP8 and AtDMP9 (*A. thaliana*); MtDMP8 and MtDMP9 (*M. truncatula*); SlDMP (*S. lycopersicum*); BnDMP1A, BnDMP2A, BnDMP1C, and BnDMP2C (*B. napus*); CsDMP (*C. sativus*); ClDMP3 (*C. lanatus*); and GmDMP1 and GmDMP2 (*G. max*). This strong phylogenetic association supports the hypothesis that the targeted knockout of *AhDMP3A*, *AhDMP3B*, *AhDMP8A*, *AhDMP8B*, *AhDMP9A*, and *AhDMP9B* may confer haploid induction capabilities in peanuts. Nevertheless, this hypothesis remains to be validated through functional studies.

### 2.3. Chromosomal Localization, Collinearity Analysis, and Selection Pressure Analysis of AhDMP Genes

**Figure 2:**
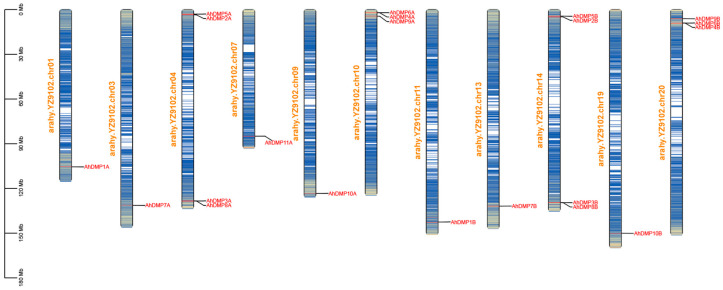
The chromosome mapping of *AhDMP* genes in the *Arachis hypogaea* genome. The physical locations of 21 *AhDMP* genes were mapped onto 11 chromosomes of *A. hypogaea* cv. YZ9102. Chromosomes are labeled with their respective names and shown in vertical orientation. *AhDMP* gene names are indicated in red, and their positions along the chromosomes are marked accordingly. Most genes are located near chromosomal ends, with a conserved distribution pattern observed between homologous chromosomes of the A and B subgenomes.

The chromosomal distribution of the 21 identified *AhDMP* genes was analyzed to explore their spatial organization and evolutionary dynamics within the *A. hypogaea* genome. These genes were mapped across eleven different chromosomes (Figure 2), predominantly localized near the distal regions, which are often associated with higher rates of recombination and gene activity. In line with the hybrid and allopolyploid origin of *A. hypogaea*, *AhDMP* genes displayed conserved collinear patterns across homologous chromosomes in the A and B subgenomes, suggesting selective retention during polyploidization, which is likely a reflection of their essential biological roles. Chromosomes Chr04 and Chr14 contained the highest number of *AhDMP* genes, indicating potential hotspots of *DMP* gene expansion. Interestingly, *AhDMP11A* was uniquely located on Chr07 of the A subgenome, with no corresponding homologous gene identified in the syntenic region of Chr17 in the B subgenome. This asymmetry may be the result of post-polyploidization gene loss or functional divergence.

**Figure 3:**
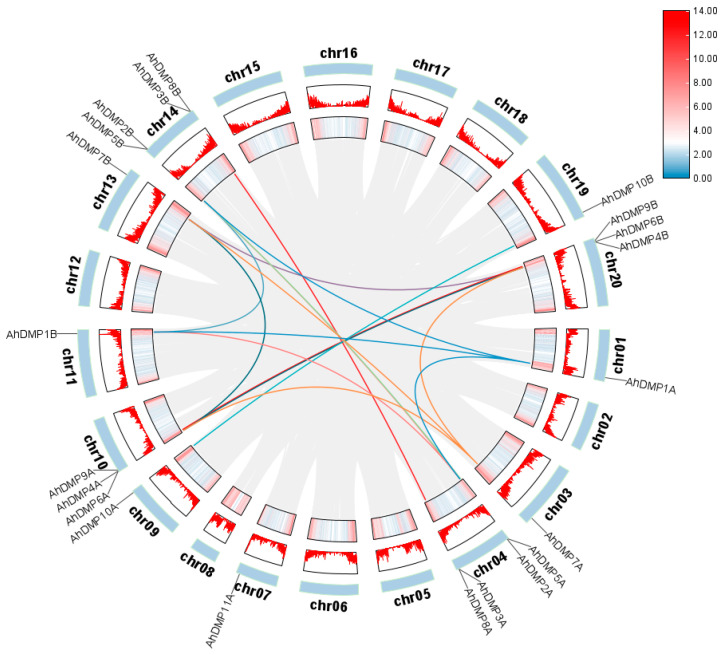
Intraspecific syntenic relationships of *AhDMP* genes in the *Arachis hypogaea* genome. Circular representation of syntenic relationships among *AhDMP* genes across the 20 chromosomes of *A. hypogaea* (AABB genome). Gene locations are labeled on the outer ring, with red histograms and heatmaps indicating gene density. Colored connecting lines within the circle indicate 17 syntenic gene pairs, highlighting conserved segmental duplications primarily between the homologous chromosomes of the A and B subgenomes.

Gene duplication serves as a fundamental force in plant genome evolution, promoting gene family expansion and functional diversification. Among the principal mechanisms of whole-genome duplication (WGD), tandem duplication, and segmental duplication, each contributes uniquely to shaping genomic architecture and complexity [31,32]. In this study, intraspecific synteny analysis revealed 17 pairs of homologous *AhDMP* genes (Table 2), and the absence of tandem duplication events suggests that WGD and segmental duplication events were the main drivers of *AhDMP* gene family amplification. These homologous pairs are primarily located on corresponding chromosomes between the A and B subgenomes (Figure 3), suggesting the conserved retention of gene duplicates following allotetraploidization in *A. hypogaea*. This syntenic pattern highlights the evolutionary stability and potential functional importance of *AhDMP* genes. Interestingly, *AhDMP11A* lacks a detectable syntenic counterpart, possibly due to the loss or divergence of its homologous gene during evolution.

**Figure 4:**
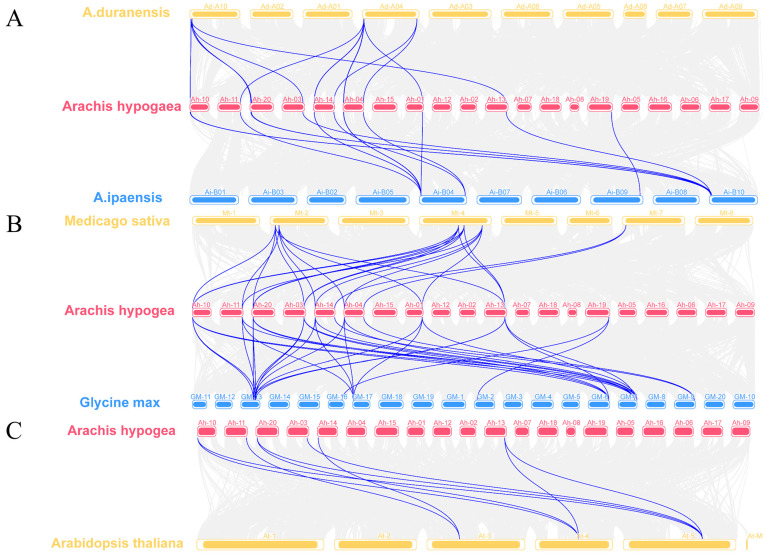
Interspecific syntenic relationships of *AhDMP* genes between *Arachis hypogaea* and five other plant species. Blue lines indicate conserved syntenic gene pairs. (**A**) Collinearity between *A. hypogaea* and its wild diploid progenitors *A. duranensis* (AA genome) and *A. ipaensis* (BB genome). (**B**) Collinearity of the *AhDMP* gene in *A. hypogaea* and related leguminous species *Medicago sativa* and *Glycine max* demonstrates the extensive conservation of *DMP* genes, suggesting shared evolutionary origins and potential functional conservation. (**C**) Collinearity of the *AhDMP* gene between *A. hypogaea* and the model species *Arabidopsis thaliana* reveals fewer conserved syntenic pairs, indicating greater evolutionary divergence.

To further elucidate the evolutionary dynamics of the *AhDMP* genes, we performed synteny analyses between *A. hypogaea* and several representative species, including its two progenitor species (*A. duranensis* and *A. ipaensis*), two other legume species (*M. truncatula* and *G. max*), and *A. thaliana* (Figure 4). Synteny analysis identified 13 conserved gene pairs between the A subgenome of *A. hypogaea* (AABB) and *A. duranensis* (AA), with 15 pairs detected between the B subgenome and *A. ipaensis* (BB), supporting the close evolutionary relationship between cultivated peanut and its diploid progenitors. Comparative analysis with other legumes revealed 22 syntenic gene pairs with *M. truncatula* and 32 gene pairs with *G. max,* which are both significantly higher than the 9 gene pairs identified in the distantly related model species *A. thaliana*. This pattern highlights the conserved evolution of *DMP* genes within the legume family and suggests the presence of shared functional constraints. Notably, *AhDMP7A*, *AhDMP6A*, *AhDMP7B*, and *AhDMP4B* each maintained at least three syntenic gene pairs with both *M. truncatula* and *G. max,* indicating a high level of conservation. These genes may have played crucial roles in the evolutionary trajectory and could be functionally significant within leguminous crops.

To assess the evolutionary pressures acting on *AhDMP* genes, we calculated nonsynonymous-to-synonymous substitution rate ratios (Ka/Ks) for both intraspecific and interspecific gene pairs. All 17 intraspecific *AhDMP* gene pairs exhibited Ka/Ks ratios below 0.5 (Table 2), indicating a strong purifying selection. Similarly, among the 66 interspecific gene pairs, 64 pairs showed Ka/Ks ratios below 0.5, with only 2 pairs falling between 0.5 and 1 (Table S1). These findings suggest that the *DMP* gene family has evolved under strong functional constraints, with purifying selection acting to maintain gene stability and conserve essential functions across both cultivated peanut and its related species.

### 2.4. Conserved Motif and Domain Analysis of AhDMP

**Figure 5:**
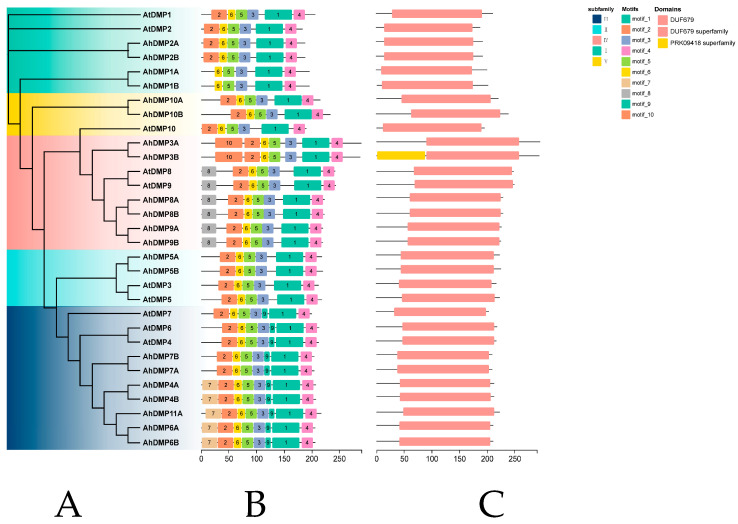
Conserved motifs and protein domain architectures in *DMP* families in *Arabidopsis thaliana* and *Archis hypogaea*. (**A**) Neighbor-joining (NJ) phylogenetic tree of *A. thaliana* (*AtDMPs*) and *A. hypogaea* (*AhDMPs*), with clades distinguished by different colors. (**B**) Conserved motif composition of all DMPs. Ten motifs (motif_1 to motif_10) are represented by colored boxes. (**C**) Predicted protein domain structures of AtDMPs and AhDMPs. Pink boxes indicate the DUF679 domain, and additional domains (e.g., PRK09418 superfamily) are labeled accordingly.

To explore potential functional divergence among *AhDMP* gene family members, we performed a comparative analysis of conserved motifs and protein domains in *A. thaliana* and *A. hypogaea* using the MEME Suite and NCBI CD-Search tools. A total of ten conserved motifs were detected across all DMPs. Individual AhDMPs contained between five and eight motifs, with AhDMP1A and AhDMP1B possessing the fewest (five) and AhDMP4A, AhDMP4B, AhDMP6A, AhDMP6B, and AhDMP11A containing the most (eight) motifs (Figure 5). Members of the same group generally shared similar motif compositions. However, AhDMP3A and AhDMP3B diverged from other Group IV members by lacking motif 8 and containing motif 10, suggesting possible functional divergence. In contrast, AhDMP8A, AhDMP8B, AhDMP9A, and AhDMP9B exhibited motif patterns identical to those of AtDMP8 and AtDMP9, implying conserved functionality across species. Several motifs exhibited group-specific distribution patterns. Motif 8 was exclusive to Group IV; motif 9 was unique to Group III; and motif 7 was restricted to AhDMP4A, AhDMP4B, AhDMP11A, AhDMP6A, and AhDMP6B, all within Group III. These findings suggest that such group-specific motifs may contribute to functional divergence within the *DMP* gene family, although further functional validation is required to confirm their biological significance.

### 2.5. Gene Structure and Cis-Acting Element Analysis of the AhDMP Gene Family

To further investigate the function of the *AhDMP* gene family in *A. hypogaea*, we examined both their gene structures and promoter regions. Structural analysis revealed that all *AhDMP* genes are intronless, and those belonging to the same phylogenetic group exhibit a strongly conserved gene structure, suggesting functional similarity.

**Figure 6:**
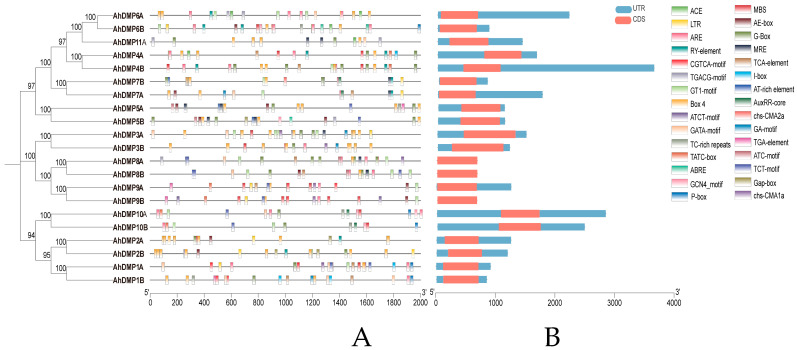
Promoter *cis*-acting element distribution and gene structure of *AhDMP* genes. (**A**) Predicted cis-acting regulatory elements within the 2000 bp upstream promoter regions of *AhDMP* genes. Different colored symbols represent distinct functional categories of regulatory elements. (**B**) Gene structure analysis of *AhDMP* genes. Red boxes represent coding sequences (CDSs), and blue boxes represent untranslated regions (UTRs). The gene models highlight variations in exon–intron organizations among gene family members.

Promoter regions, as key determinants of transcriptional initiation and spatiotemporal gene expression, were further analyzed for *cis*-acting regulatory elements. This investigation uncovered a diverse array of *cis*-acting elements associated with hormonal signaling, stress, light responsiveness, and developmental processes across the promoter regions of 21 *AhDMP* genes (Figure 6). Notably, light-responsive elements were the most prevalent, accounting for 54% of all identified elements and distributed broadly across the promoters, such as ACE, and the GT1-motif. The presence of seed-specific elements such as the RY-element and GCN4_motif in *AhDMP10A* and *AhDMP5B* suggests their potential role in integrating light signals with seed development. Moreover, every *AhDMP* promoter harbored at least one hormone-responsive element, reflecting their possible involvement in diverse hormonal pathways. These included elements responsive to salicylic acid (TCA-element), gibberellin (TATC-box, P-box), methyl jasmonate (CGTCA-motif, TGACG-motif), abscisic acid (ABRE), and auxin (AuxRR-core). In addition, a number of elements associated with abiotic stress were identified, such as drought-inducible MBS, defense and stress-responsive TC-rich repeats, cold-responsive LTR, anaerobic-inducible ARE, and heat-responsive AT-rich elements (Table S2). Collectively, these results indicate that *AhDMP* gene expression is intricately regulated by both phytohormones and environmental factors.

### 2.6. Expression Profiles of Peanut AhDMP Genes Across Different Tissues

**Figure 7:**
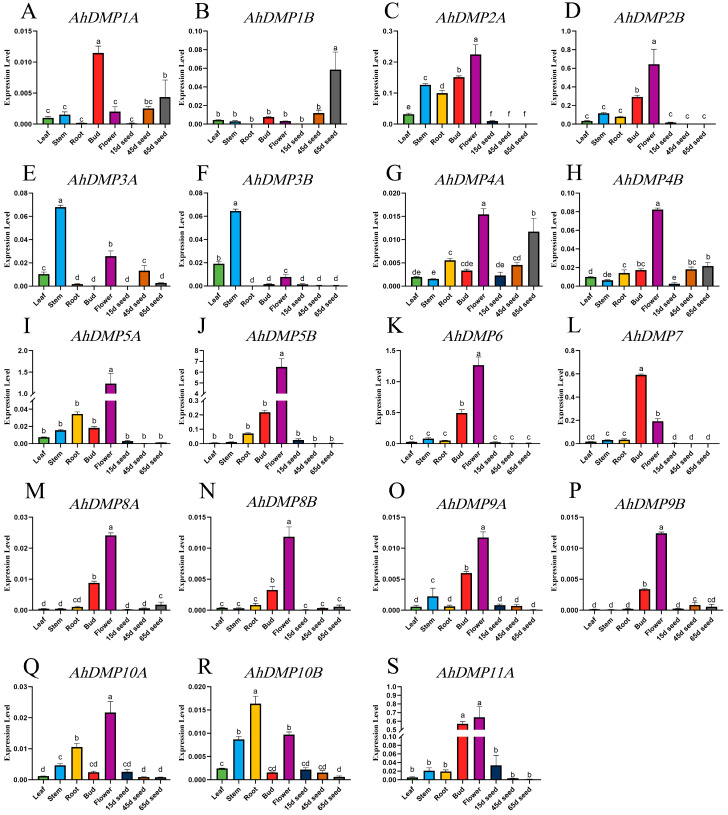
Tissue-specific expression profiles of *AhDMP* genes in peanuts. (**A**–**S**) The relative expression levels of 21 *AhDMP* genes were analyzed across eight different tissues, including roots, stems, leaves, buds, flowers, 15d seeds, 45d seeds, and 65d seeds, using quantitative reverse transcriptase PCR (RT-qPCR). Expression data were normalized against the internal reference gene *AhADH3*. Error bars indicate the standard deviation of triplicate biological replicates. Different letters above the bars denote significant differences (*p* < 0.05) among tissues.

To further elucidate the potential biological functions of *AhDMP* gene family members, we conducted an analysis of their tissue-specific expression patterns in *A. hypogaea* (Table S3). The results revealed that homologous gene pairs generally display similar expression profiles, reflecting possible functional redundancy or co-regulation. In line with previous reports that haploid induction genes are predominantly expressed in reproductive organs [24], most *AhDMP* genes exhibited their highest expression levels in floral tissues (Figure 7). Although *AhDMP1A* and *AhDMP1B* exhibited low expressions overall, *AhDMP1A* and *AhDMP7* were more actively transcribed in buds rather than in flowers, suggesting their potential role in early flower development. In contrast, *AhDMP1B* showed elevated expression in 65d seeds, suggesting its possible involvement in seed maturation. Notably, *AhDMP10B* demonstrated peak expression in roots, pointing to a possible root-specific function. *AhDMP2A* and *AhDMP2B* were highly expressed in flowers and exhibited minimal transcriptional activity in seeds. In contrast, *AhDMP3A* and *AhDMP3B* showed their highest expression in stems, with negligible expression in roots and buds, suggesting tissue-specific divergence within this group. Furthermore, *AhDMP4A* and *AhDMP4B* exhibited progressively increasing expressions throughout seed development, implying their role in seed development. The remaining *AhDMP* genes were predominantly expressed in flowers, aligning with their presumed roles in reproduction. Phylogenetic analysis revealed that *AhDMP3A*, *AhDMP3B*, *AhDMP8A*, *AhDMP8B*, *AhDMP9A*, and *AhDMP9B* cluster within the same clade as *ZmDMP*, *AtDMP8,* and *AtDMP9,* all of which have been experimentally validated as haploid induction genes. Among these, *AhDMP8A*, *AhDMP8B*, *AhDMP9A,* and *AhDMP9B* exhibited significantly elevated expressions in floral tissues, suggesting that they may possess haploid-inducing capabilities in peanuts. Based on this expression profile, we hypothesize that the targeted suppression or knockout of these four genes may facilitate haploid induction. In contrast, although *AhDMP3A* and *AhDMP3B* belong to the same phylogenetic group, their preferential expression in stems implies a divergence in function, rendering them less likely to be involved in haploid induction.

### 2.7. Subcellular Localization of DMPs in Arachis hypogaea

**Figure 8:**
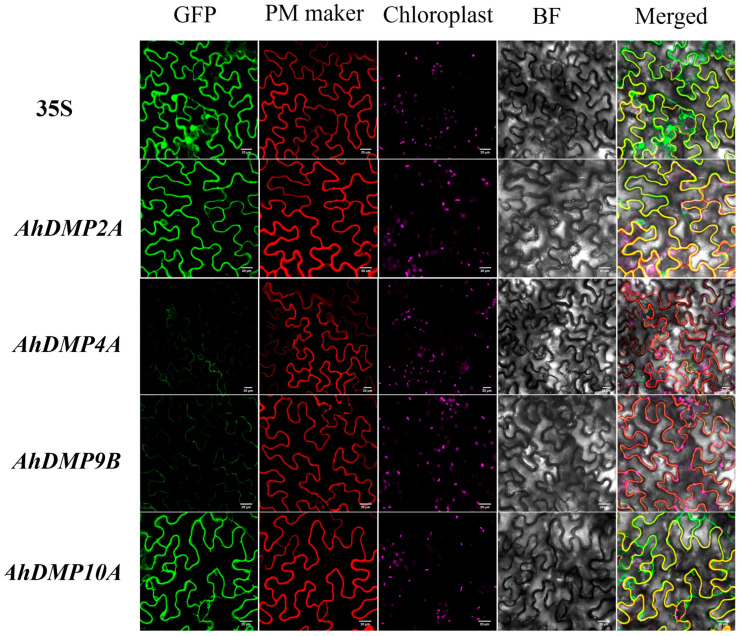
The subcellular localization of selected AhDMPs in *Nicotiana benthamiana* epidermal cells. The transient expression of GFP-tagged AhDMP2A, AhDMP4A, AhDMP9B, and AhDMP10A fusion proteins was observed in *N. benthamiana* leaves using confocal laser scanning microscopy. Positive control experiments were conducted using the 35S::eGFP construct. Columns from left to right show GFP fluorescence, plasma membrane markers (red), chloroplast (magenta), a bright-field image, and merged channels. Scale bars = 20 μm.

To investigate where DMPs are localized within *A. hypogaea* cells, we constructed expression vectors and transiently expressed them in *N. benthamiana* leaves. Confocal microscopy analysis revealed that AhDMP2A, AhDMP4A, AhDMP9B, and AhDMP10A exhibited strong co-localization with a plasma membrane marker protein (Figure 8) [33], corroborating the in silico predictions obtained from the Plant-Ploc database. By contrast, diffuse fluorescence was observed in both the nucleus and plasma membrane in the empty vector control. Notably, the plasma membrane localization of AhDMP9B mirrors the subcellular distribution patterns of its *A. thaliana* orthologs [23,27], suggesting an evolutionarily conserved membrane-associated function. These findings imply that certain AhDMPs may exert their biological roles at the plasma membrane, potentially mediating signal transduction or membrane-associated cellular processes.

## 3. Discussion

DMPs are a class of plant-specific proteins predominantly localized to cellular membranes. Recent studies have increasingly uncovered the functional significance of several *DMP* family members. For instance, *StDMP2* has been shown to enhance resistance against *Phytophthora infestans* in potato [11], while a point mutation within the first TM domain of *ZmDMP* has been shown to trigger haploid induction. Moreover, *ZmDMP* homologs across diverse dicotyledonous species have also been proven to induce haploidy [22]. A systematic analysis of the *A. hypogaea* genome led to the identification of 21 *DMP* gene family members, which exceeds the numbers found in its diploid ancestors, *A. duranensis* and *A. ipaensis*. This expansion is likely a consequence of polyploidization events during the evolution of allotetraploid peanuts, which can give rise to functional diversification or pseudogene formation. All identified *AhDMP* genes were intronless, possessed the conserved DUF679 domain, and encoded proteins with 2–4 predicted TMs. Each *AtDMP* gene from *A. thaliana* was found to have at least one homologous counterpart in peanuts, and conserved motif structures were observed among *AhDMP* and *AtDMP* genes within the same clades, indicating a high degree of functional conservation. This observation supports the hypothesis that the targeted knockout of *AhDMP* homologs corresponding to *AtDMP8* and *AtDMP9* may confer haploid induction potential in peanuts. Promoter analysis further revealed a wide distribution of cis-acting elements responsive to light, stress, and hormones across *AhDMP* gene promoters. These were followed by a high abundance of elements associated with hormone signaling and environmental cues.

Phylogenetic analysis revealed that *AhDMP3A*, *AhDMP3B*, *AhDMP8A*, *AhDMP8B*, *AhDMP9A,* and *AhDMP9B* were clustered within Group Ⅳ alongside *AtDMP8*, *AtDMP9,* and *ZmDMP,* which are genes previously reported to possess haploid-inducing functions. These *DMP* genes are typically characterized by a strong expression in floral tissues [24]. Consistent with this pattern, our quantitative reverse transcriptase PCR (RT-qPCR) results demonstrated that *AhDMP8A*, *AhDMP8B*, *AhDMP9A,* and *AhDMP9B* exhibit flower-specific expression patterns similar to *AtDMP8* and *AtDMP9*. Based on their phylogenetic relationships and expression profiles, we hypothesize that these four *AhDMP* genes may participate in double fertilization, and their targeted knockout could potentially generate haploid-inducing peanut lines. Conversely, *AhDMP3A* and *AhDMP3B,* although phylogenetically related to haploid-inducing functional *DMP* genes, did not display the same floral expression pattern, suggesting that they may have distinct biological functions. More broadly, the majority of *AhDMP* genes exhibited elevated expression in flowers, indicating their potential involvement in reproductive processes. However, several members demonstrated distinct tissue-specific expression; for example, *AhDMP1B* is most highly expressed in 65d seeds, *AhDMP3A* and *AhDMP3B* are most highly expressed in stems, and *AhDMP10B* is most highly expressed in roots. These patterns imply that while many *AhDMP* genes are associated with reproductive development, others may have diversified functional roles in vegetative development or stress responses.

Subcellular localization is a key factor influencing protein function, and fusion reporter assays are commonly used to visualize protein distribution within cells. In this study, we constructed expression vectors by fusing the coding sequences of AhDMP2A, AhDMP4A, AhDMP9B, and AhDMP10A with the enhanced green fluorescent protein (eGFP) reporter gene. Following transient expression in *N. benthamiana*, protein localization was analyzed using confocal microscopy. The results demonstrated that all four AhDMPs were localized to the plasma membrane. While this confirms their membrane association, the precise biological functions these proteins perform at the plasma membrane remain to be elucidated through further functional studies.

Peanut has a relatively narrow genetic base and exhibits unique reproductive characteristics that pose significant obstacles to artificial pollination, thereby hindering the development of improvements [17,18]. In comparison to conventional breeding methods, doubled haploid technology offers substantial advantages, including shortened breeding cycles, accelerated germplasm improvement, and reduced seed production costs [19]. Given that the generation of homozygous inbred lines is a fundamental objective of most plant breeding programs, developing a robust and dependable method for haploid induction has long been a central focus of research.

The first haploid-inducer line in maize, Stock6, was identified in 1950. When crossed with various genotypes, it produces haploids at a frequency of 2–3%, leading to its designation as a haploid-inducing line [34]. Since then, Stock6-derived inducer lines have become a major method for haploid production in maize. In 2017, the molecular basis of the Stock6-induced haploid formation was elucidated through the identification of the key gene *ZmPLA1*/*MTL*/*NLD,* independently cloned by several research groups [35,36,37]. Functional studies have demonstrated that the knockout of *ZmPLA1*/*MTL*/*NLD* homologs enables the development of haploid-inducer lines in other cereals, including rice and wheat [38,39,40,41]. The broad conservation of *MTL* across monocotyledonous species highlights its potential for broad applications in cereal crops. However, its *A. thaliana* homolog *AtPLP2* is expressed only in vegetative tissues and does not support haploid induction [42]. Notably, *ZmDMP* was the first haploid induction gene identified outside the Stock6 lineage. When co-mutated with *MTL*, *ZmDMP* synergistically enhances haploid induction frequency, increasing it by 5–6 fold [22]. In dicotyledonous species, haploid induction systems have been successfully established by editing *ZmDMP* homologs. For example, in *A. thaliana*, the simultaneous knockout of *AtDMP8* and *AtDMP9* resulted in a haploid induction efficiency of 2.1 ± 1.1% [23]. Additional studies have demonstrated successful *DMP*-mediated haploid inductions in *M. sativa* [27], *N. tabacum*, and *B. napus* [24], with reported efficiencies ranging from 0.29 to 0.82%, 1.1 to 2.4%, and 1.2%, respectively. Although *DMP*-based haploid inducer lines currently exhibit relatively low efficiencies, the conserved nature of floral expression patterns of *DMP* genes across monocotyledonous and dicotyledonous species suggests their broad applicability. Despite these advances, no studies to date have reported haploid induction via *ZmDMP* homologs in peanuts. This presents an untapped opportunity for leveraging DMP-based systems in peanut breeding programs.

## 4. Materials and Methods

### 4.1. Identification and Analysis of the DMP Members

The genome and GFF annotation files for *A. hypogaea* cv. *YZ9102* were retrieved from the Figshare repository (https://doi.org/10.6084/m9.figshare.26551309.v1, accessed on 4 December 2024) [43]. Genomic data for the wild diploid peanut *A. duranensis* (v1.SWBf) and *A. ipaensis* (v1.bxj8) were obtained from the PeanutBase database (https://www.peanutbase.org/, accessed on 11 April 2024). Additional reference genomes, including *M. truncatula* (v4.0), *G. max* (v2.1), *N. tabacum* (v2.0), *Z. mays* (v5.0), and *A. thaliana* (TAIR, v10), were sourced from the Ensembl Plants database (https://plants.ensembl.org/index.html, accessed on 11 April 2024). Genomic sequences of *G. hirsutum* (v3.0), *B. napus* (ZS11.v0), *S. lycopersicum* (v5.0), *C. lanatus* (v2.5), *C. sativus* (v1.0), and *S. tuberosum* (v6.1) were acquired from the Phytozome database (https://phytozome-next.jgi.doe.gov/, accessed on 11 April 2024). The hidden Markov model (HMM) profile PF05078, corresponding to the DUF679 domain characteristic of the *DMP* gene family, was obtained from the Pfam database (http://pfam.xfam.org/, accessed on 11 April 2024). Protein databases of all selected species were scanned using the Simple HMM Search tool from TBtools (v2.315) with PF05078 as the query profile (*E*-value < 1 × 10^−5^) [44]. Additionally, the published *A. thaliana* DMP sequences were used as queries in BLASTp searches against the peanut protein database to identify candidate sequences [23]. To improve the precision of the results, candidate genes shared by both approaches were selected. The physicochemical properties of the identified AhDMPs were predicted using ExPASy (https://web.expasy.org/protparam/, accessed on 31 March 2025) [45], while subcellular localization was predicted with Plant-Ploc (http://www.csbio.sjtu.edu.cn/bioinf/plant/, accessed on 31 March 2025) [46]; the DeepTMHMM tool (https://dtu.biolib.com/DeepTMHMM, accessed on 31 March 2025) was utilized to predict the number of transmembrane domains [47].

### 4.2. Multiple Sequence Alignment and Phylogenetic Analysis of DMP Genes

Multiple sequence alignment of DMP orthologs from 14 plant species was performed using DNAMAN 2.0 and ClustalW [48,49]. An NJ phylogenetic tree was constructed using MEGA 11 software with 1000 bootstrap replicates [50]. The resulting tree was visualized and refined using Chiplot (https://www.chiplot.online/, accessed on 16 April 2025) [51].

### 4.3. Conserved Domain and Motif Analysis of DMP Genes

Evolutionarily conserved domains in AhDMPs were identified using the NCBI Conserved Domina Database (CD-Search) (Home-Conserved Domains-NCBI, accessed on 15 April 2025) [52]. Conserved motifs among AhDMPs were further analyzed using the MEME suite under the 10-motif with default parameterization (http://meme-suite.org/tools/meme, accessed on 15 April 2025) [53].

### 4.4. Chromosomal Localization, Gene Duplication, and Selective Pressure Analyses

Based on the genome annotation data of *A. hypogaea* cv. YZ9102, the physical positions of *AhDMP* genes were determined and mapped onto chromosomes. The chromosomal distribution of the *AhDMP* genes was visualized using TBtools. Gene duplication events, including both intra- and interspecific duplications, were detected through MCScanX analysis implemented within the TBtools platform. To assess evolutionary pressures, duplicated *DMP* gene pairs were then subjected to Ka and Ks substitution rate calculation using the Ka/Ks Calculator module in TBtools.

### 4.5. Analysis of Promoter Region and Gene Structure in AhDMP Genes

The primary gene structures, including the exon and intron organization of *AhDMPs,* were obtained from the genome annotation analysis of the *A. hypogaea* cultivar YZ9102. To investigate potential regulatory elements, 2000 bp upstream sequences from the start codon of the *AhDMP* gene were extracted as putative promoter regions using TBtools. These promoter sequences were then analyzed using the PlantCARE database (https://bioinformatics.psb.ugent.be/webtools/plantcare/html/, accessed on 15 April 2025) to predict *cis*-acting regulatory elements, including their types, locations, and numbers [54].

### 4.6. qRT-PCR Analysis of AhDMP Genes

Following the manufacturer’s guidelines, total RNA was extracted from peanut cv. YH9326 using the MiniBEST Kit (Takara, Tokyo, Japan). RNA was isolated from eight tissue types: the root, stem, leaf, bud, flower, 15d seed, 45d seed, and 65d seed. First-strand cDNA was reverse-transcribed from isolated RNA, serving as the template for RT-qPCR.

Primers for *AhDMPs* were designed based on their CDSs and verified through Sanger sequencing. RT-qPCR analysis deployed ChamQ Universal SYBR qPCR Master Mix (Vazyme, Nanjing, China). *AhADH3* was used as the internal reference gene. All reactions were conducted in triplicate, and relative gene expression levels were calculated using the 2^−Δ*Ct*^ method [55,56]. The primers used for the expression analysis are listed in Table S4.

### 4.7. Subcellular Localization of AhDMPs

The coding sequences of *AhDMP2A*, *AhDMP4A*, *AhDMP5B*, *AhDMP9B*, and *AhDMP10A* were cloned into the pCAMBIA1300-eGFP vector via homologous recombination. The stop codon (TAA) was removed to ensure in-frame C-terminal fusion with the eGFP tag. Recombinant constructs were transformed into *Agrobacterium tumefaciens* GV3101. Agrobacterial suspensions containing the constructs were mixed in a 1:1 ratio with a plasma membrane marker solution and infiltrated into *N. benthamiana* leaves at the 4–6 leaf stage. Subsequently, subcellular localization was observed via confocal laser scanning microscopy. Primer sequences for vector construction are documented in Table S5.

## 5. Conclusions

This study represents the first comprehensive characterization of the *DMP* gene family in cultivated peanuts, shedding light on their evolutionary trajectories, expression patterns, and potential functional roles. Phylogenetic and expression analysis identified *AhDMP8A, AhDMP8B, AhDMP9A,* and *AhDMP9B* as the strongest candidates for haploid induction, owing to their strong sequence homology and similar floral expression profiles closely resembling those of the known haploid-inducing genes *AtDMP8* and *AtDMP9*. In contrast, other *AhDMP* family members displayed distinct tissue-specific expression patterns, such as enrichment in roots, stems, or leaves, suggesting that they may play broader roles in peanut development and physiology beyond reproduction. These conclusions were derived from an integrative approach combining genome-wide identification, phylogenetic, synteny analyses, expression profiling via RT-qPCR, and subcellular localization studies. Notably, transient expression assays in *N. benthamiana* confirmed that representative AhDMPs localize to the plasma membrane, aligning with their proposed roles in fertilization-related processes. Collectively, these findings establish a valuable foundation for future functional studies of *DMP* genes in peanuts and point to promising targets for the development of a double haploid breeding system.

## Figures and Tables

**Table 1:** The physicochemical characterization of *DMP* gene family members in *Arachis hypogaea* cv. YZ9102.

Chr	Gene ID	Gene Name	Protein Length (aa)	Molecular Weight (MW)/kDa	Isoelectric Point (pi)	Transmembrane Domains	Subcellular Location
Chr01	*g2643.t1*	*AhDMP1A*	196	21.34	9.17	4	Plasma Membrane
Chr01	*g9634.t1*	*AhDMP7A*	204	22.64	5.41	2	Plasma Membrane
Chr04	*g11185.t1*	*AhDMP2A*	188	20.30	7.68	4	Plasma Membrane
Chr04	*g13490.t1*	*AhDMP3A*	289	31.35	6.42	4	Plasma Membrane/Extracellular
Chr04	*g11140.t1*	*AhDMP5A*	218	23.41	8.64	3	Plasma Membrane/Extracellular
Chr04	*g13491.t1*	*AhDMP8A*	223	24.68	8.52	4	Plasma Membrane
Chr07	*g23068.t1*	*AhDMP11A*	217	24.05	4.99	4	Plasma Membrane
Chr09	*g29711.t1*	*AhDMP10A*	215	24.40	6.88	4	Plasma Membrane
Chr10	*g30131.t1*	*AhDMP4A*	207	22.30	4.87	3	Plasma Membrane
Chr10	*g30130.t1*	*AhDMP6A*	206	22.64	4.85	4	Plasma Membrane
Chr10	*g30358.t1*	*AhDMP9A*	220	24.26	8.73	4	Plasma Membrane
Chr11	*g35860.t1*	*AhDMP1B*	196	21.41	9.3	4	Plasma Membrane
Chr13	*g43762.t1*	*AhDMP7B*	204	22.64	5.41	2	Plasma Membrane
Chr14	*g45489.t1*	*AhDMP2B*	188	20.36	6.88	4	Plasma Membrane
Chr14	*g48076.t1*	*AhDMP3B*	287	31.16	6.94	4	Plasma Membrane/Extracellular
Chr14	*g45442.t1*	*AhDMP5B*	220	23.71	8.64	3	Plasma Membrane/Extracellular
Chr14	*g48077.t1*	*AhDMP8B*	223	24.68	8.52	4	Plasma Membrane
Chr19	*g66369.t2*	*AhDMP10B*	233	26.45	6.28	4	Plasma Membrane
Chr20	*g67987.t1*	*AhDMP4B*	207	22.46	4.8	4	Plasma Membrane
Chr20	*g67988.t1*	*AhDMP6B*	206	22.68	4.98	4	Plasma Membrane
Chr20	*g67734.t1*	*AhDMP9B*	220	24.23	8.73	4	Plasma Membrane

**Table 2:** Intraspecific gene pairs and Ka/Ks analysis in *Arachis hypogaea*.

Gene Name	Gene Name	Duplication Type	Ka	Ks	Ka/Ks
*AhDMP1A*	*AhDMP2A*	WGD or Segmental	0.386466	0.879288	0.439522
*AhDMP1A*	*AhDMP1B*	WGD or Segmental	0.009065	0.028289	0.320457
*AhDMP1A*	*AhDMP2B*	WGD or Segmental	0.380863	0.846162	0.450107
*AhDMP1B*	*AhDMP2B*	WGD or Segmental	0.389245	0.875567	0.444564
*AhDMP2A*	*AhDMP1B*	WGD or Segmental	0.386909	0.905388	0.42734
*AhDMP2A*	*AhDMP2B*	WGD or Segmental	0.009438	0.044957	0.209928
*AhDMP3A*	*AhDMP3B*	WGD or Segmental	0.016748	0.063586	0.263386
*AhDMP5A*	*AhDMP5B*	WGD or Segmental	0.014868	0.058225	0.255349
*AhDMP6A*	*AhDMP7B*	WGD or Segmental	0.156265	1.180603	0.132361
*AhDMP6A*	*AhDMP4B*	WGD or Segmental	0.167732	0.587088	0.285701
*AhDMP7A*	*AhDMP6A*	WGD or Segmental	0.156328	1.14425	0.13662
*AhDMP7A*	*AhDMP7B*	WGD or Segmental	0	0.014035	0
*AhDMP7A*	*AhDMP4B*	WGD or Segmental	0.206464	0.966381	0.213647
*AhDMP7B*	*AhDMP4B*	WGD or Segmental	0.206379	0.968674	0.213053
*AhDMP8A*	*AhDMP8B*	WGD or Segmental	0.003908	0.032792	0.119161
*AhDMP9A*	*AhDMP9B*	WGD or Segmental	0.008008	0.039012	0.205272
*AhDMP1A*	*AhDMP1B*	WGD or Segmental	0.00381	0.047801	0.079708

## Data Availability

The original contributions presented in this study are included in the article. Further inquiries can be directed to the corresponding author(s).

## Supplementary Materials

The following supporting information can be downloaded at https://www.mdpi.com/article/10.3390/ijms26157243/s1.

## References

[1] Xicluna J., Lacombe B., Dreyer I., Alcon C., Jeanguenin L., Sentenac H., Thibaud J.-B., Chérel I. (2007). Increased Functional Diversity of Plant K+ Channels by Preferential Heteromerization of the Shaker-like Subunits AKT2 and KAT2. J. Biol. Chem..

[2] Chen Y., Heazlewood J.L. (2021). Organellar Proteomic Profiling to Analyze Membrane Trafficking Pathways. Trends Plant Sci..

[3] Chen Y., Weckwerth W. (2020). Mass Spectrometry Untangles Plant Membrane Protein Signaling Networks. Trends Plant Sci..

[4] Mori T., Kawai-Toyooka H., Igawa T., Nozaki H. (2015). Gamete Dialogs in Green Lineages. Mol. Plant.

[5] Yamada K., Osakabe Y., Mizoi J., Nakashima K., Fujita Y., Shinozaki K., Yamaguchi-Shinozaki K. (2010). Functional Analysis of an *Arabidopsis thaliana* Abiotic Stress-Inducible Facilitated Diffusion Transporter for Monosaccharides. J. Biol. Chem..

[6] Cyprys P., Lindemeier M., Sprunck S. (2019). Gamete Fusion Is Facilitated by Two Sperm Cell-Expressed DUF679 Membrane Proteins. Nat. Plants.

[7] Kasaras A., Melzer M., Kunze R. (2012). Arabidopsis Senescence-Associated Protein DMP1 Is Involved in Membrane Remodeling of the ER and Tonoplast. BMC Plant Biol..

[8] Olvera-Carrillo Y., Van Bel M., Van Hautegem T., Fendrych M., Van Durme M., Huysmans M., Šimášková M., Buscaill P., Rivas S., Coll N.S. (2015). A Conserved Core of PCD Indicator Genes Discriminates Developmentally and Environmentally Induced Programmed Cell Death in Plants. Plant Physiol..

[9] Kasaras A., Kunze R. (2010). Expression, Localisation and Phylogeny of a Novel Family of Plant-specific Membrane Proteins. Plant Biol..

[10] Takahashi T., Mori T., Ueda K., Yamada L., Nagahara S., Higashiyama T., Sawada H., Igawa T. (2018). The Male Gamete Membrane Protein DMP9/DAU2 Is Required for Double Fertilization in Flowering Plants. Development.

[11] Bi W., Chen Y., Song Y., Liu J., Zhao T., Sun C., Qin J., Tu Z., Li Y., Wang X. (2025). Potato DMP2 Positively Regulates Plant Immunity by Modulating Endoplasmic Reticulum Homeostasis. J. Integr. Plant Biol..

[12] Ahmad Z., Tian D., Li Y., Aminu I.M., Tabusam J., Zhang Y., Zhu S. (2024). Characterization, Evolution, Expression and Functional Divergence of the DMP Gene Family in Plants. Int. J. Mol. Sci..

[13] Ma Y., Liu H., Wang J., Zhao G., Niu K., Zhou X., Zhang R., Yao R. (2024). Genomic Identification and Expression Profiling of DMP Genes in Oat (*Avena sativa*) Elucidate Their Responsiveness to Seed Aging. BMC Genom..

[14] Zhu S., Wang X., Chen W., Yao J., Li Y., Fang S., Lv Y., Li X., Pan J., Liu C. (2021). Cotton DMP Gene Family: Characterization, Evolution, and Expression Profiles during Development and Stress. Int. J. Biol. Macromol..

[15] Nawade B., Bosamia T.C., Lee J.H., Jang J.H., Lee O.R. (2023). Genome-Wide Characterization of the Soybean DOMAIN OF UNKNOWN FUNCTION 679 Membrane Protein Gene Family Highlights Their Potential Involvement in Growth and Stress Response. Front. Plant Sci..

[16] Sarvamangala C., Gowda M.V.C., Varshney R.K. (2011). Identification of Quantitative Trait Loci for Protein Content, Oil Content and Oil Quality for Groundnut (*Arachis hypogaea* L.). Field Crops Res..

[17] Smith B.W. (1950). Arachis hypogaea. Aerial flower and subterranean fruit. Am. J. Bot..

[18] Bertioli D.J., Seijo G., Freitas F.O., Valls J.F.M., Leal-Bertioli S.C.M., Moretzsohn M.C. (2011). An Overview of Peanut and Its Wild Relatives. Plant Genet. Res..

[19] Jacquier N.M.A., Gilles L.M., Pyott D.E., Martinant J.-P., Rogowsky P.M., Widiez T. (2020). Puzzling out Plant Reproduction by Haploid Induction for Innovations in Plant Breeding. Nat. Plants.

[20] Yongping X., Yunhuan Z., Yingduo G., Zhaocong C. (2022). The first report on one haploid plant in cultivated peanut using anther culture technique. Chin. J. Oil Crop Sci..

[21] Croser J.S., Lülsdorf M.M., Davies P.A., Clarke H.J., Bayliss K.L., Mallikarjuna N., Siddique K.H.M. (2006). Toward Doubled Haploid Production in the Fabaceae: Progress, Constraints, and Opportunities. Crit. Rev. Plant Sci..

[22] Zhong Y. (2019). Mutation of ZmDMP Enhances Haploid Induction in Maize. Nat. Plants.

[23] Zhong Y. (2020). A DMP-Triggered In vivo Maternal Haploid Induction System in the Dicotyledonous Arabidopsis. Nat. Plants.

[24] Establishment of a Dmp Based Maternal Haploid Induction System for Polyploid *Brassica napus* and *Nicotiana tabacum*. https://onlinelibrary.wiley.com/doi/epdf/10.1111/jipb.13244.

[25] Chen X., Li Y., Ai G., Chen J., Guo D., Zhu Z., Zhu X., Tian S., Wang J., Liu M. (2023). Creation of a Watermelon Haploid Inducer Line via ClDMP3-Mediated Single Fertilization of the Central Cell. Hortic. Res..

[26] Long L., Feng Y.-M., Shang S.-Z., Zhao J.-R., Hu G.-Y., Xu F.-C., Song C.-P., Jin S.-X., Gao W. (2023). In Vivo Maternal Haploid Induction System in Cotton. Plant Physiol..

[27] Wang N., Xia X., Jiang T., Li L., Zhang P., Niu L., Cheng H., Wang K., Lin H. (2022). In Planta Haploid Induction by Genome Editing of DMP in the Model Legume Medicago Truncatula. Plant Biotechnol. J..

[28] Yin S., Li S., Sun L., Shi K., Fan S., Liu X., Ren H. (2024). Mutating the Maternal Haploid Inducer Gene CsDMP in Cucumber Produces Haploids in Planta. Plant Physiol..

[29] Zhang J., Yin J., Luo J., Tang D., Zhu X., Wang J., Liu Z., Wang P., Zhong Y., Liu C. (2022). Construction of Homozygous Diploid Potato through Maternal Haploid Induction. aBIOTECH.

[30] Zhong Y., Yang M., Cheng D., Liu J., Han Q., Liu C., Qi X., Yan T., Teng L., Xv C. Mutation of GmDMP Genes Triggers Haploid Induction in Soybean.

[31] Cannon S.B., Mitra A., Baumgarten A., Young N.D., May G. (2004). The Roles of Segmental and Tandem Gene Duplication in the Evolution of Large Gene Families in *Arabidopsis thaliana*. BMC Plant Biol..

[32] Vision T.J., Brown D.G., Tanksley S.D. (2000). The Origins of Genomic Duplications in Arabidopsis. Science.

[33] Linster E., Layer D., Bienvenut W.V., Dinh T.V., Weyer F.A., Leemhuis W., Brünje A., Hoffrichter M., Miklankova P., Kopp J. (2020). The Arabidopsis Nα -acetyltransferase NAA60 Locates to the Plasma Membrane and Is Vital for the High Salt Stress Response. New Phytol..

[34] Coe E.H. (1959). A Line of Maize with High Haploid Frequency. Am. Nat..

[35] Liu C., Li X., Meng D., Zhong Y., Chen C., Dong X., Xu X., Chen B., Li W., Li L. (2017). A 4-Bp Insertion at ZmPLA1 Encoding a Putative Phospholipase A Generates Haploid Induction in Maize. Mol. Plant.

[36] Kelliher T., Starr D., Richbourg L., Chintamanani S., Delzer B., Nuccio M.L., Green J., Chen Z., McCuiston J., Wang W. (2017). MATRILINEAL, a Sperm-Specific Phospholipase, Triggers Maize Haploid Induction. Nature.

[37] Gilles L.M., Khaled A., Laffaire J., Chaignon S., Gendrot G., Laplaige J., Bergès H., Beydon G., Bayle V., Barret P. (2017). Loss of Pollen-specific Phospholipase NOT LIKE DAD Triggers Gynogenesis in Maize. EMBO J..

[38] Wang C., Liu Q., Shen Y., Hua Y., Wang J., Lin J., Wu M., Sun T., Cheng Z., Mercier R. (2019). Clonal Seeds from Hybrid Rice by Simultaneous Genome Engineering of Meiosis and Fertilization Genes. Nat. Biotechnol..

[39] Liu H., Wang K., Jia Z., Gong Q., Lin Z., Du L., Pei X., Ye X. (2020). Efficient Induction of Haploid Plants in Wheat by Editing of TaMTL Using an Optimized Agrobacterium-Mediated CRISPR System. J. Exp. Bot..

[40] Cheng Z., Sun Y., Yang S., Zhi H., Yin T., Ma X., Zhang H., Diao X., Guo Y., Li X. (2021). Establishing in Planta Haploid Inducer Line by Edited SiMTL in Foxtail Millet (*Setaria italica*). Plant Biotechnol. J..

[41] Tang H., Qiu Y., Wang W., Yu M., Chang Y., Han Z., Du L., Lin Z., Wang K., Ye X. (2023). Development of a Haploid Inducer by Editing HvMTL in Barley. J. Genet. Genom..

[42] La Camera S., Geoffroy P., Samaha H., Ndiaye A., Rahim G., Legrand M., Heitz T. (2005). A Pathogen-Inducible Patatin-like Lipid Acyl Hydrolase Facilitates Fungal and Bacterial Host Colonization in Arabidopsis. Plant J..

[43] Wang X., Sun Z., Qi F., Zhou Z., Du P., Shi L., Dong W., Huang B., Han S., Pavan S. (2025). A Telomere-to-Telomere Genome Assembly of the Cultivated Peanut. Mol. Plant.

[44] Chen C., Wu Y., Li J., Wang X., Zeng Z., Xu J., Liu Y., Feng J., Chen H., He Y. (2023). TBtools-II: A “One for All, All for One” Bioinformatics Platform for Biological Big-Data Mining. Mol. Plant.

[45] Gasteiger E. (2003). ExPASy: The Proteomics Server for in-Depth Protein Knowledge and Analysis. Nucleic Acids Res..

[46] Chou K.-C., Shen H.-B. (2008). Cell-PLoc: A Package of Web Servers for Predicting Subcellular Localization of Proteins in Various Organisms. Nat. Protoc..

[47] Hallgren J., Tsirigos K.D., Pedersen M.D., Almagro Armenteros J.J., Marcatili P., Nielsen H., Krogh A., Winther O. DeepTMHMM Predicts Alpha and Beta Transmembrane Proteins Using Deep Neural Networks.

[48] Thompson J.D., Higgins D.G., Gibson T.J. (1994). CLUSTAL W: Improving the Sensitivity of Progressive Multiple Sequence Alignment through Sequence Weighting, Position-Specific Gap Penalties and Weight Matrix Choice. Nucleic Acids Res..

[49] Zhu X., Leng X., Sun X., Mu Q., Wang B., Li X., Wang C., Fang J. (2015). Discovery of Conservation and Diversification of miR171 Genes by Phylogenetic Analysis Based on Global Genomes. Plant Genome.

[50] Tamura K., Stecher G., Kumar S. (2021). MEGA11: Molecular Evolutionary Genetics Analysis Version 11. Mol. Biol. Evol..

[51] Xie J., Chen Y., Cai G., Cai R., Hu Z., Wang H. (2023). Tree Visualization By One Table (tvBOT): A Web Application for Visualizing, Modifying and Annotating Phylogenetic Trees. Nucleic Acids Res..

[52] Wang J., Chitsaz F., Derbyshire M.K., Gonzales N.R., Gwadz M., Lu S., Marchler G.H., Song J.S., Thanki N., Yamashita R.A. (2023). The Conserved Domain Database in 2023. Nucleic Acids Res..

[53] Bailey T.L., Johnson J., Grant C.E., Noble W.S. (2015). The MEME Suite. Nucleic Acids Res..

[54] Lescot M. (2002). PlantCARE, a Database of Plant Cis-Acting Regulatory Elements and a Portal to Tools for in Silico Analysis of Promoter Sequences. Nucleic Acids Res..

[55] Livak K.J., Schmittgen T.D. (2001). Analysis of relative gene expression data using real-time quantitative PCR and the 2^− ΔΔCT^ method. Methods.

[56] Shah K., Zhang W., Zhou H., Cheng B., Zhang Z., Yang Z., Moale C., Kamanova S., Han M., Ren X. (2022). Identification of MdMED Family, Key Role of MdMED81, and Salicylic Acid at the Right Time of Year Triggers MdMED81 to Induce Flowering in Malus Domestica. Sci. Hortic..

